# Supplementary material for: Decreasing peptide deformylase activity is a beneficial strategy for increasing formaldehyde resistance in Methylobacterium extorquens
Source: bioRxiv. 2026 Apr 21:2026.04.16.718930. Preprint. [Version 1] doi: 10.64898/2026.04.16.718930 (PMC13131673; doi:10.64898/2026.04.16.718930)
Supplement: 1 [file NIHPP2026.04.16.718930V1-supplement-1.pdf]

bioRxiv preprint doi: https://doi.org/10.64898/2026.04.16.718930; this version posted April 21, 2026. The copyright holder for this preprint (which was not certified by peer review) is the author/funder, who has granted bioRxiv a license to display the preprint in perpetuity. It is made available under aCC-BY 4.0 International license.

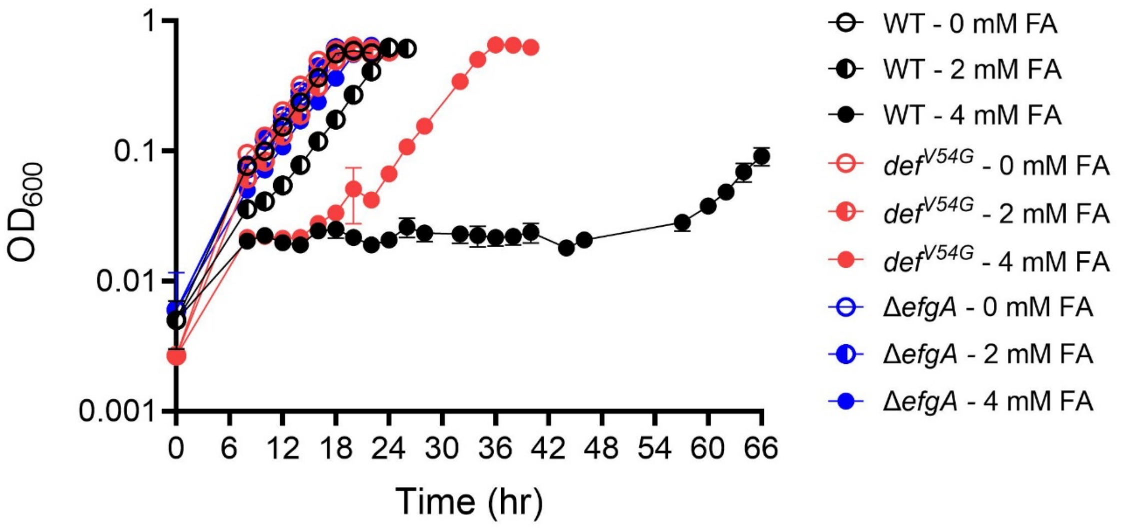

Figure S1: Formaldehyde growth assay with WT, *def*^{evo}, and Δ*efgA*.

WT, Δ*efgA*, and *def*^{evo} strains were grown in MP with 3.5 mM succinate and 0, 2, or 4 mM formaldehyde. *def*^{evo} and Δ*efgA* have increased resistance to formaldehyde at all concentrations tested. The error bars represent the standard deviation of the mean for three biological replicates.

bioRxiv preprint doi: https://doi.org/10.64898/2026.04.16.718930; this version posted April 21, 2026. The copyright holder for this preprint (which was not certified by peer review) is the author/funder, who has granted bioRxiv a license to display the preprint in perpetuity. It is made available under aCC-BY 4.0 International license.

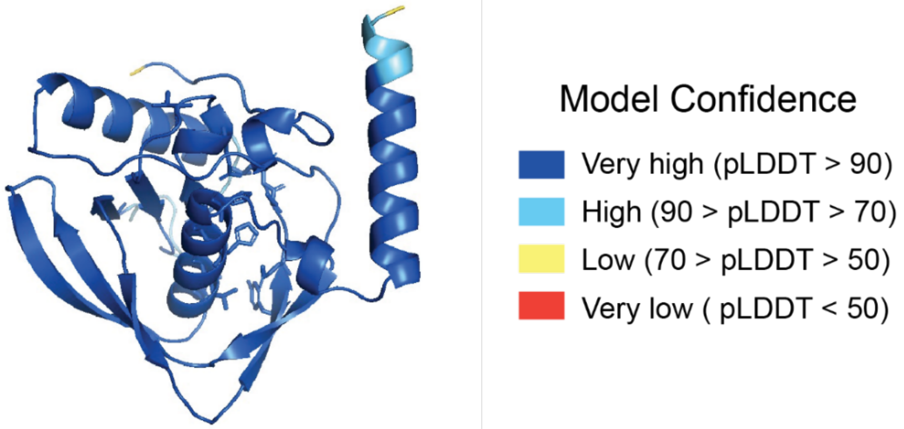

Figure S2: The structural model for PDF^{WT} has high confidence.

The pLDDT scores reported by AlphaFold are shown. 156 of the 171 residues have scores over 90. None of the residues have scores below 50.

bioRxiv preprint doi: https://doi.org/10.64898/2026.04.16.718930; this version posted April 21, 2026. The copyright holder for this preprint (which was not certified by peer review) is the author/funder, who has granted bioRxiv a license to display the preprint in perpetuity. It is made available under aCC-BY 4.0 International license.

A

```
CLUSTAL 2.1 multiple sequence alignment


mex_Mext_1636       MTVRPLVILP----DAQLRLTSEPVAAVTDEIRTLARDMIETMYDAPGVGLAAIQIGVAK
ccs_CCNA_00273      MAIRRILTVDNAADLATLKKISTPVEAVTDELRALMDDMLETMYDAPGIGLAAVQVGEPV
eco_b3287           MSVLQVLHIP----DERLRKVAKPVEEVNAEIQRIVDDMFETMYAEEGIGLAATQVDIHQ
nme_NMB0110         MALLNILQYP----DERLHTVAKPVEQVDERIRKLIADMFETMYESRGIGLAATQVDVHE
ppu_PP_0068         MAILNILEFP----DPRLRTLAKPVTEFDDALRQLIDDMFETMYEAPGIGLAATQVNVHK
hpy_HP_0793         MALLEIIHYP----SKILRTISKEVVSFDSKLHQQLDDMHETMIASEGIGLAAIQVGLPL
bfr_BF1684          -MILPIYVYG----QPVLRQVAEDITVDYPNLKELIENMFETMDHADGVGLAAPQIGLPI
                      :  :           *:  :  :      ::    :* ***    *:**** *:.

mex_Mext_1636       RVVTID---TSKDE--NAKNPTVYLNPEIVWVSEEKRVYDEGCLSIPEFYGEVERPDRVR
ccs_CCNA_00273      RVIVMD---LAREG--EDKAPRYFVNPEILASSEDLQGYEEGCLSVPEYYDEVERPSKVT
eco_b3287           RIIVID---VSEN----RDERLVLINPELLEKSGET-GIEEGCLSIPEQRALVPRAEKVK
nme_NMB0110         RVVVMD---LTED----RSEPRVFINPVIVEKDGET-TYEEGCLSVPGIYDTVTRAERVK
ppu_PP_0068         QVVVMD---LSED----RSEPRVFINPSVEELTHDMGQYQEGCLSVPGFYENVDRPLRVR
hpy_HP_0793         RMLIIN---LPQEDGVQHKEDCLEIINPKFIETGGSMMYREGCLSVPGFYEEVERFEKVK
bfr_BF1684          RVVVINLDVLSEDYPEYKDFRKAYINAHIDVVEGEEVSMEEGCLSLPGIHESVKRGSKIH
                    ::: ::    ..:     .     :               *****:*     * *  ::

mex_Mext_1636       VRYMNLDGQIVEQEADGLLATCLQHEIDHLNGVLFIDHLSKLKRDRVMKKFTKAAKRDAA
ccs_CCNA_00273      LRYMNYQGETVVEEAEGLFAVCIQHEMDHLEGVLFIDHLSRLRRDRAMAKVKKARRAA--
eco_b3287           IRALDRDGKPFELEADGLLAICIQHEMDHLVGKLFMDYLSPLKQQRIRQKVEKLDRLKAR
nme_NMB0110         VEALNEKGEKFTLEADGLLAICVQHELDHLMGIVFVERLSQLKQGRIKTKLKKRQKHTI-
ppu_PP_0068         VKAQDRDGKPFELECEGLLAVCVQHEFDHLNGKLFVDYLSQLKRDRIKKKLEKQHRQQA-
hpy_HP_0793         IEYQNRFAEVKVLEASELLAVAIQHEIDHLNGVLFVDKLSILKRKKFEKELKELQKKQKH
bfr_BF1684          VRYMDENFVEHNEVVEGFLARVMQHEFDHLDGKMFIDHISPLRKQMIKGKLNTMLKGKAR
                    :.  :          . ::*  :***:*** * :*:: :* *::     :.    :

mex_Mext_1636       ---------
ccs_CCNA_00273      ---------
eco_b3287           A--------
nme_NMB0110         ---------
ppu_PP_0068         ---------
hpy_HP_0793         E--------
bfr_BF1684          SSYKMKQVK
```

B

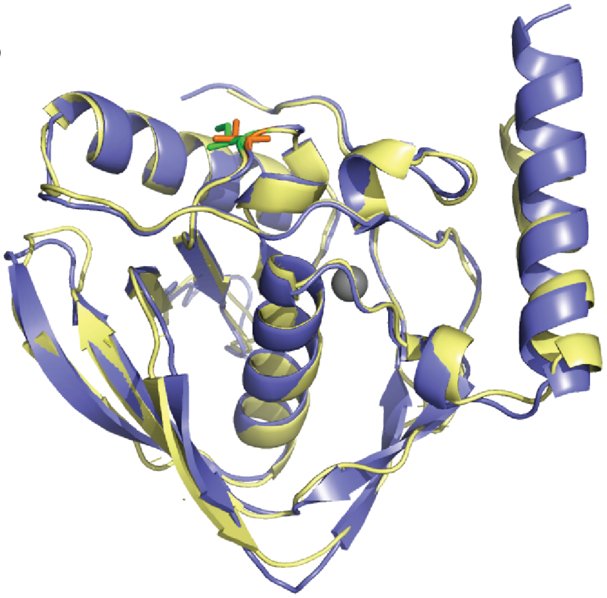

Figure S3: Sequence alignment of PDF from phylogenetically diverse Gram-negative bacteria.

bioRxiv preprint doi: https://doi.org/10.64898/2026.04.16.718930; this version posted April 21, 2026. The copyright holder for this preprint (which was not certified by peer review) is the author/funder, who has granted bioRxiv a license to display the preprint in perpetuity. It is made available under aCC-BY 4.0 International license.

A) A Clustal Omega (47) alignment of PDF from *M. extorquens* PA1 (mex), *Caulobacter vibrioides* NA1000 (ccs), *E. coli* K-12 MG1655 (eco), *Neisseria meningitidis* MC58 (serogroup B) (nme), *Pseudomonas putida* KT2440 (ppu), *Helicobacter pylori* 26695 (hpy), and *Bacteroides fragilis* YCH46 (bfr). Conservation of residues is indicated when identical (*), strongly similar (:), or weakly similar (.). Green shading indicates position of Valine 54 in *M. extorquens*. B) *M. extorquens* PDF model (blue) aligned to solved structure of *E. coli* PDF (PDB accession: 1DFF) (yellow). The *M. extorquens* V54 residue is highlighted in orange. The *E. coli* I53 residue is highlighted in green.

bioRxiv preprint doi: https://doi.org/10.64898/2026.04.16.718930; this version posted April 21, 2026. The copyright holder for this preprint (which was not certified by peer review) is the author/funder, who has granted bioRxiv a license to display the preprint in perpetuity. It is made available under aCC-BY 4.0 International license.

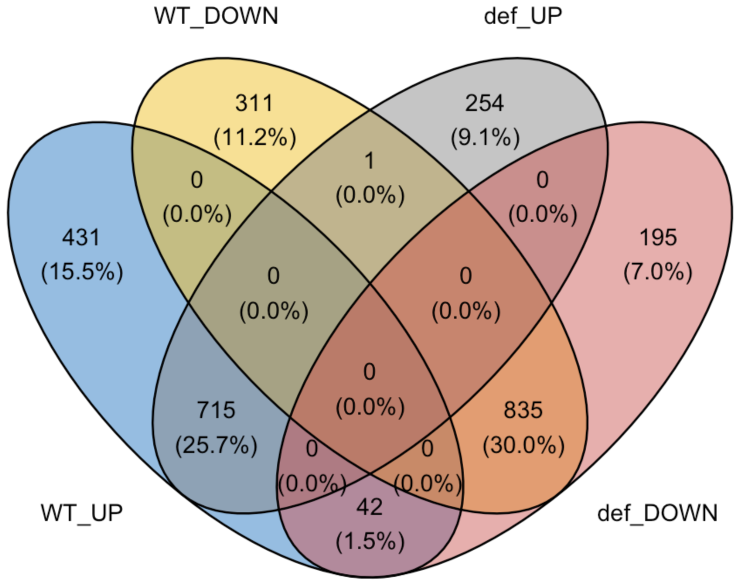

Figure S4: Venn diagram comparing gene expression changes of WT and *def*^{evo} strains under formaldehyde shock.

Comparison of differentially expressed genes in WT and *def*^{evo} after exposure to 5 mM formaldehyde for 10 min. Data are for expression changes where FDR ≤ 0.01.

bioRxiv preprint doi: https://doi.org/10.64898/2026.04.16.718930; this version posted April 21, 2026. The copyright holder for this preprint (which was not certified by peer review) is the author/funder, who has granted bioRxiv a license to display the preprint in perpetuity. It is made available under aCC-BY 4.0 International license.

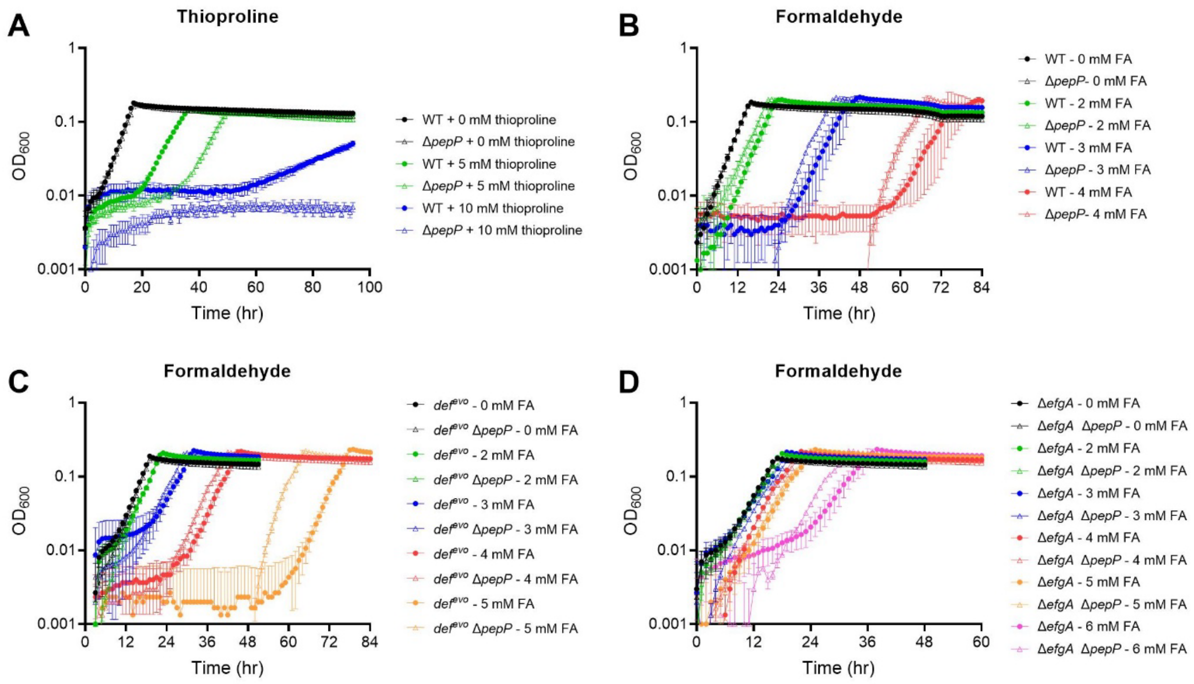

Figure S5: Thioproline does not drive formaldehyde toxicity in *M. extorquens.*

A) WT and Δ*pepP* (JB1062, *Mext_4480*) strains were grown in MP with 3.5 mM succinate and 0, 5, or 10 mM thioproline. *pepP* encodes a Xaa-Pro aminopeptidase that hydrolyzes peptides in which the second amino acid is either proline or thioproline. In *E. coli,* PepP (EC:3.4.11.9) cleaves thioproline-containing peptides to mitigate formaldehyde-induced protein damage, and mutants lacking *pepP* have increased sensitivity to formaldehyde and thioproline (37). This data demonstrates that PepP has a conserved role for mitigating thioproline stress in *M. extrorquens*. B) WT and Δ*pepP* strains were grown in MP with 3.5 mM succinate and 0, 2, 3, or 4 mM formaldehyde. The Δ*pepP* mutant grew modestly better than WT in the presence of exogenous formaldehyde, suggesting that loss of PepP-mediated peptide degradation actually alleviates formaldehyde toxicity in *M. extorquens*. A similar increase in formaldehyde resistance was seen when *pepP* was deleted in C) the *def^evo* mutant background (JB1116) and D) the Δ*efgA* mutant

bioRxiv preprint doi: https://doi.org/10.64898/2026.04.16.718930; this version posted April 21, 2026. The copyright holder for this preprint (which was not certified by peer review) is the author/funder, who has granted bioRxiv a license to display the preprint in perpetuity. It is made available under aCC-BY 4.0 International license.

background (JB1117). These findings suggest that while thioproline itself is toxic in *M. extorquens*, thioproline-containing peptides are not a driver of formaldehyde toxicity.
